# GPR37 and its neuroprotective mechanisms: bridging osteocalcin signaling and brain function

**DOI:** 10.3389/fcell.2024.1510666

**Published:** 2024-11-20

**Authors:** Xuepeng Bian, Yangping Wang, Weijie Zhang, Changlin Ye, Jingjing Li

**Affiliations:** ^1^ Department of Rehabilitation, School of International Medical Technology, Shanghai Sanda University, Shanghai, China; ^2^ Physical Education College, Shanghai University, Shanghai, China; ^3^ School of Exercise and Health, Shanghai University of Sport, Shanghai, China

**Keywords:** osteocalcin, GPR37, brain function, inflammation, stress response, neuroprotection

## Abstract

Osteocalcin (OCN) is a hormone secreted by osteoblasts and has attracted widespread attention for its role in regulating brain function. Clinical studies indicate a positive correlation between levels of circulating OCN and cognitive performance. Indeed, lower circulating OCN has been detected in various neurodegenerative diseases (NDs), while OCN supplementation under certain conditions may improve cognitive function. GPR37, a G protein-coupled receptor, has recently been identified as a receptor for OCN. It exhibits distinct expression patterns across various brain regions and cell types, potentially influencing its functional roles within the brain. Research indicates that GPR37 regulates neuronal migration, cell proliferation, differentiation, and myelination. Furthermore, GPR37 has been shown to mitigate inflammation and apoptosis through various mechanisms, exerting neuroprotective effects. However, its regulatory influence on brain function exhibits inconsistency, highlighting a duality in its actions. Therefore, this review thoroughly summarizes the roles and mechanisms of GPR37 in modulating cellular physiological activities and its involvement in immune responses, stress reactions, and neuroprotection. It aims to enhance the understanding of how GPR37 modulates brain function and facilitate the identification of novel therapeutic targets or strategies for related diseases.

## 1 Introduction

Osteocalcin (OCN), a protein composed of 44–56 amino acids, is secreted by osteoblasts (Komori, 2020; Nowicki and Jakubowska-Pietkiewicz, 2024) and was initially considered primarily involved in bone mineralization. Subsequent research has revealed that OCN can circulate through the bloodstream and enter various tissues and organs, such as skeletal muscle and liver. In these regions, OCN regulates insulin sensitivity, glucose and lipid metabolism, and skeletal muscle function (Komori, 2020; Nowicki and Jakubowska-Pietkiewicz, 2024). Furthermore, OCN crosses the blood-brain barrier (BBB) and exerts regulatory effects on the central nervous system (CNS), particularly regarding cognitive function and mood regulation (Shan et al., 2023). Several clinical studies have demonstrated a positive correlation between circulating levels of OCN and cognitive function. In various neurodegenerative diseases (NDs), such as Alzheimer’s disease (AD) and Parkinson’s disease (PD), lower circulating levels of OCN are observed (Hou et al., 2021; Liu et al., 2023). Mice deficient in OCN exhibit deficits in spatial learning and hippocampus-dependent memory (Oury et al., 2013). The supplementation of OCN can potentially improve spatial learning and memory by reducing amyloid-beta (Aβ) deposition and gliosis, elevating levels of monoamine neurotransmitters, and promoting neuroplasticity within the hippocampus and cortex (Shan et al., 2023).

The functions of OCN are contingent upon its receptors. To date, three OCN receptors have been identified in mammals: GPR37 (G protein-coupled receptor 37), GPR158, and GPRC6A, all classified as G protein-coupled receptors (Karsenty, 2023). These receptors exhibit distinct regional distributions and fulfill various functions within the body. This review focuses on GPR37, the most recently identified central receptor for OCN. Notably, GPR37 exhibits high expression in the brain and is significantly associated with the development and prognosis of various CNS diseases. The deficiency of GPR37 can result in dopaminergic neuronal damage and disrupt long-term potentiation (LTP) (Hertz et al., 2019; Zhang et al., 2020a). GPR37 may also exhibit bidirectional effects in certain physiological phenomena. In a stroke model, GPR37 negatively correlates with serum inflammatory factor levels (McCrary et al., 2019; Zhang et al., 2022). Conversely, in lipopolysaccharide (LPS)-induced inflammation models, the expression of GPR37 is significantly elevated, further activating glial cells and exacerbating the inflammatory response (Qian et al., 2022).

Given the complex and uncertain roles of GPR37 in various functions, along with the incomplete understanding of its regulations in the CNS, this review aims to summarize the roles and mechanisms of GPR37 to enrich the “bone-brain axis” theory further and offer new targets for the treatment of NDs.

## 2 Identification and distribution of GPR37

**FIGURE 1:**
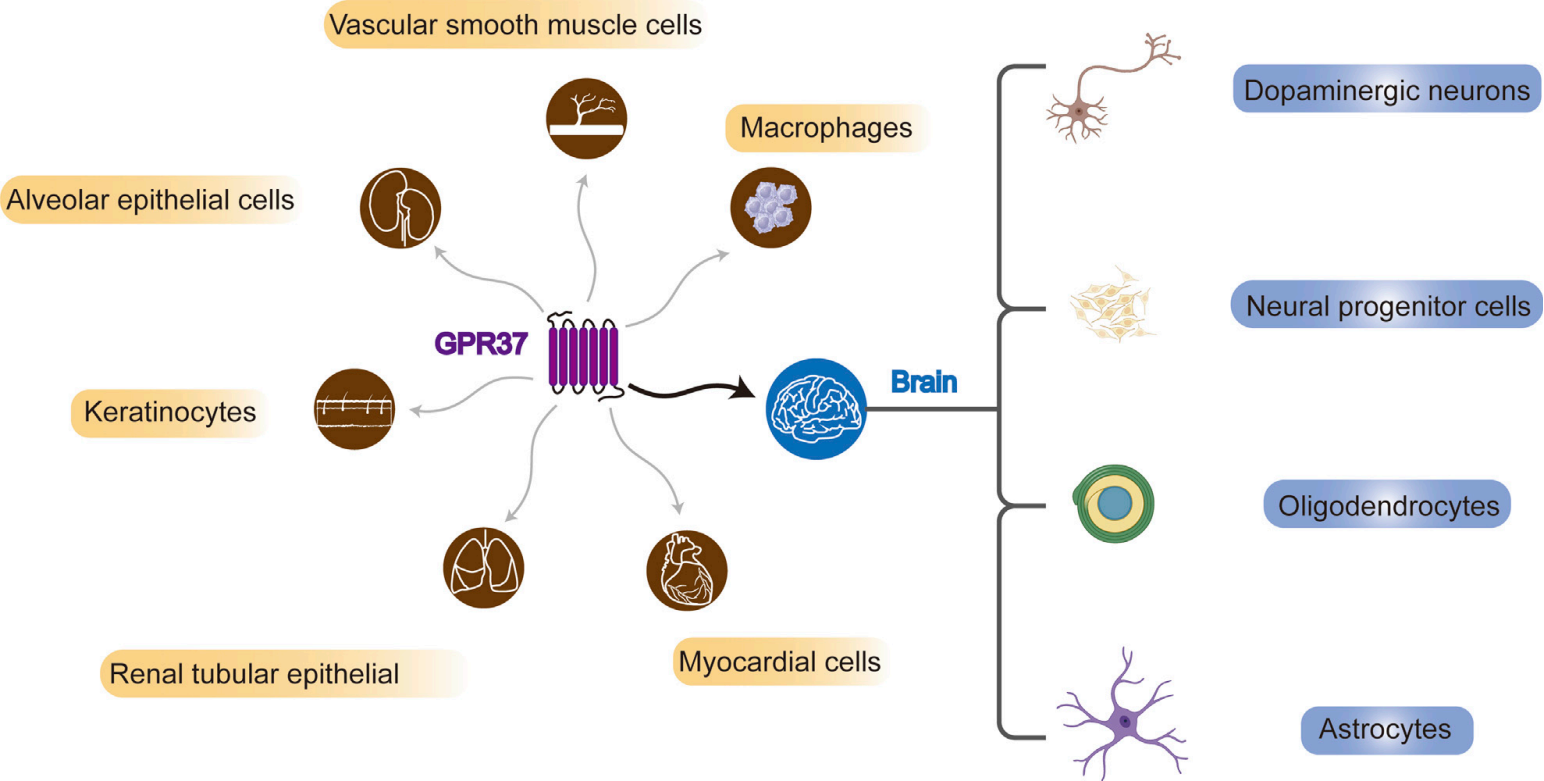
Systemic and brain distribution of GPR37. GPR37 is expressed in various cell types, including vascular smooth muscle cells, macrophages, alveolar and renal tubular epithelial cells, keratinocytes, and myocardial cells. In the brain, it is found in dopaminergic neurons, neural progenitors, oligodendrocytes, and astrocytes.

In 1997, GPR37 was identified by analyzing cDNA expression sequence tags from the human frontal cortex, utilizing RACE-PCR technology to study neuropeptide-specific receptor genes (Marazziti et al., 1997). Subsequent research has revealed that GPR37 is expressed in multiple brain regions of the CNS (Yang et al., 2016; Mouhi et al., 2022) and different types of cells, including substantia nigra dopaminergic neurons (Imai et al., 2001; Morato et al., 2021), neural progenitor cells (NPCs) (Berger et al., 2017; Owino et al., 2021), oligodendrocytes (OLs), and astrocytes (Bang et al., 2018). However, in microglia, GPR37 is unidentified (Bang et al., 2018). The expression of GPR37 may vary even in the same type of cells, which may depend on the stage of cell development. For example, GPR37 is highly expressed in mature OLs but not in oligodendrocyte precursor cells (OPCs) (Yang et al., 2016) (Figure 1).

Previous studies have demonstrated that OCN binds specifically to GPR37 but not to its homolog GPR37L1, as confirmed by affinity assays and immunoprecipitation techniques (Qian et al., 2021). Further investigations reveal that OCN is involved in myelination via GPR37. Exogenous injection of OCN in wild-type (WT) mice significantly decreases the levels of myelin-associated proteins—proteolipid protein 1 (PLP1) and myelin basic protein (MBP) in the corpus callosum and spinal cord. Notably, this effect is absent in GPR37−/− mice, indicating that OCN’s actions are mediated through GPR37 (Qian et al., 2021). In primary cultured OLs, inhibition of GPR37 using shRNA or antibodies significantly attenuates the OCN-induced reduction of PLP1 and MBP, whereas silencing GPR37L1 does not affect this downregulation (Qian et al., 2021). These *in vivo* and *in vitro* findings demonstrate that OCN exerts specific effects through GPR37, establishing a distinct ligand-receptor relationship between them.

## 3 Central regulatory functions of GPR37

The role of GPR37 can be traced back to studies on its homolog, SCGPR1, in chicken embryos. SCGPR1 demonstrates significant developmental expression in the neural tube, forebrain, midbrain, and spinal cord. This experiment suggests that GPR37 may be expressed in varying temporal and spatial patterns depending on the developmental stage and needs of the organism as it progresses from an embryo to an adult (Odani et al., 2007). The involvement of OCN in embryonic development offers insights into the developmental regulation of GPR37 expression. During pregnancy, maternal OCN crosses the placental barrier and enters the embryonic bloodstream, which plays a neuroprotective role by preventing apoptosis of hippocampal neurons (Oury et al., 2013). OCN levels synchronize with cognitive changes from growth and development to aging. Maternal and embryonic OCN contribute to establishing and maintaining body homeostasis in newborns and adult offspring, influencing brain development (Oury et al., 2013; Correa Pinto Junior et al., 2024). With aging, the decline in bone mass and OCN levels, along with a progressive decrease in the activity of critical molecules essential for cellular functions, such as nicotinamide adenine dinucleotide (NAD) and NRF2, collectively contribute to cognitive decline (Nishimoto et al., 1985; Silva-Palacios et al., 2018; Fania et al., 2019).

A deficiency in OCN contributes to a range of peripheral metabolic disorders and markedly reduces the expression of genes related to glucose metabolism in the brain. With advancing age, OCN−/− mice develop insulin resistance and glucose intolerance, while supplementation with OCN mitigates these metabolic disturbances (Ferron et al., 2008; Ferron et al., 2012; Zhang et al., 2020b; Paracha et al., 2024). Dysregulation of peripheral glucose metabolism is closely associated with central insulin resistance (Guo et al., 2020). Impaired insulin signaling in the brain—particularly involving the IRS/PI3K/Akt pathway—often exacerbates the pathogenesis of NDs (Dewanjee et al., 2022). These disruptions are associated with profound impairments in learning and memory during adulthood (Oury et al., 2013; Correa Pinto Junior et al., 2024). These findings highlight that maintaining optimal maternal skeletal health and adequate OCN levels during pregnancy may be critical strategies for ensuring physiological homeostasis in offspring and reducing the risk of neurodevelopmental disorders.

Furthermore, there appears to be a reciprocal interaction between brain development and bone formation during embryogenesis. Fetal chondrocytes produce OCN and differentiate into osteoblasts only when co-cultured with brain tissue, indicating a tissue-specific response (Groot et al., 1994). Additionally, the Wnt/β-catenin signaling pathway, pivotal in bone formation, shares overlapping mechanisms with GPR37-mediated signaling pathways involved in neuronal physiology (Jiang et al., 2014; Berger et al., 2017).

### 3.1 Cellular physiological activities

Research on olfactory ensheathing cells (OECs), a specialized type of glial cell primarily located in the olfactory bulb, has confirmed the pivotal role of GPR37 in facilitating neuronal migration and supporting the regeneration and repair of olfactory neurons. Treatment of primary OECs and embryonic cultures containing olfactory regions with the GPR37 inhibitor Macitentan significantly reduces the migration of gonadotropin-releasing hormone (GnRH) neurons and OECs. Conversely, the GPR37 agonist TX14A directly promotes the migration of GnRH neurons (Saadi et al., 2019). These functions of GPR37 were also validated in GPR37−/− mice, where GPR37 knockout resulted in reduced migration capacity of OECs and GnRH cells (Saadi et al., 2019). The impact of GPR37 on cell migration may be linked to reduced Akt phosphorylation or decreased RhoA-GTPase activity in OECs, which disrupts cytoskeletal reorganization and impairs GnRH cell migration (Saadi et al., 2019).

In megalencephalic leukoencephalopathy with subcortical cysts (MLC), GPR37 preserves the stability of intercellular connections by negatively regulating the expression and function of glial MLC1 and glial cell adhesion molecule (GlialCAM), thereby ensuring normal cell adhesion and signal transmission (Pla-Casillanis et al., 2022). In NPCs, knocking down GPR37 reduces the expression of doublecortin (Dcx), a neuronal marker, and the number of terminally differentiated Microtubule-associated protein 2 (MAP2)-positive cells, a marker of mature neurons. At the same time, increasing the expression of chondroitin sulfate proteoglycan 4 (Cspg4), a microglial marker (Massey et al., 2008). These findings demonstrate the crucial role of GPR37 in neuronal and glial differentiation and neurogenesis. OCN/GPR37 is involved in the differentiation of NPCs, primarily through alterations in Wnt signaling (Berger et al., 2017). Wnt signaling is more active in younger individuals and declines significantly with age, showing an age-dependent reduction (Inestrosa et al., 2020). Activating the Wnt/β-catenin pathway can prevent Aβ-induced damage to brain endothelial cells, promote BBB repair (Wang et al., 2022), and enhance hippocampal synaptic plasticity (Hu et al., 2019). Inhibition of Wnt signaling disrupts the expression of genes closely associated with the differentiation, such as vimentin (VIM), leading to excessive activation of glial cells, which interferes with neurite extension and synaptic plasticity (Pebworth et al., 2021). The changes in VIM are analogous to the perspective that molecular drivers of AD vary with age: compared to normal aging, VIM is significantly enriched in elderly patients with AD. Furthermore, the increase in VIM is more pronounced in younger AD patients than in their older counterparts (Panizza and Cerione, 2024). Excessive activation of GPR37 has been implicated in aberrant cell proliferation, particularly in tumor cells. Research indicates that GPR37 is highly expressed in gliomas, where it plays a crucial role in promoting tumor cell proliferation and migration. Its overexpression is correlated with poor clinical outcomes and is linked to the activation of critical oncogenic signaling pathways, including the PI3K-Akt and Ras pathways. Conversely, silencing GPR37 has been shown to suppress these malignant behaviors (Liang et al., 2023). In cultured human glioma U251 cells, GPR37 expression is significantly upregulated after 2 days. This phenomenon correlates with a decreased proportion of cells in the G1/G0 phase and an increased proportion in the S and G2/M phases, thus driving accelerated cell proliferation. This proliferation is further supported by a marked increase in phosphorylated Akt (Ser473) levels (Zhang and Wang, 2018).

The OCN/GPR37 signaling pathway also maintains myelin homeostasis (Smith et al., 2017; Qian et al., 2021). The absence of OCN could lead to excessive myelination in the CNS, characterized by the abundant expression of MBP and PLP1, along with an increased number of OLs. The underlying mechanism may involve the regulation of OCN on the expression of myelin-associated gene Myrf, which is a crucial transcription factor for OLs myelination and myelin maintenance. This regulation may inhibit OPCs differentiation into mature OLs (Qian et al., 2021). This process may be closely related to the effects of GPR37 on maintaining low-density lipoprotein receptor-related protein 6 (LRP6) levels and Wnt signaling in NPCs. Research indicates that the knockdown of GPR37 in NPCs leads to decreased levels of LRP6 and a reduction in the expression of Sp5, a target gene of Wnt. Furthermore, in LRP6-deficient HEK293 cells, neither GPR37 nor GPR37-1TM (the N-terminal domain of GPR37) can activate Wnt signaling unless LRP6 is reintroduced, which subsequently reactivates Wnt signaling (Berger et al., 2017). Additionally, GPR37 can promote OLs differentiation and myelination through ERK signaling (Yang et al., 2016). The influence of GPR37 on OLs differentiation is also modulated by the zinc finger transcription factor Zfp488, Nk homology domain protein Nkx2.2, and Sox10 (Schmidt et al., 2024).

**FIGURE 2:**
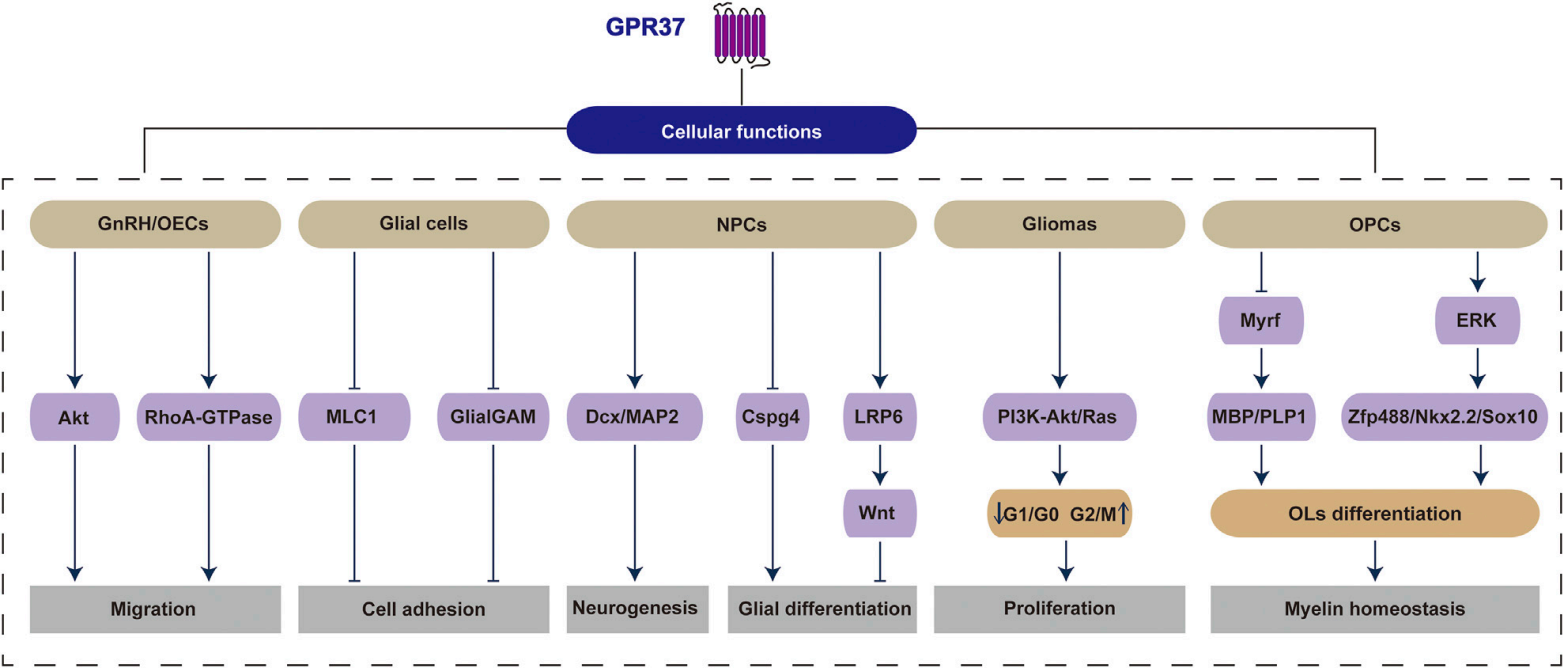
GPR37 regulates a range of physiological and pathological activities within cells. Specifically, GPR37 influences critical cellular functions across various cell types, including migration in GnRH/OECs, cell adhesion in glial cells, neurogenesis and glial differentiation in NPCs, proliferation in gliomas, as well as OLs differentiation and myelin homeostasis in OPCs.

GPR37 exerts a significant and broad regulatory influence on various cellular activities within the CNS. It involves cell proliferation, migration, differentiation, and myelination processes through diverse signaling pathways and molecular mechanisms. However, its dual role as a therapeutic target under different physiological and pathological conditions warrants further investigation (Figure 2).

### 3.2 Inflammation and immune responses

GPR37 is a crucial factor closely associated with inflammation and immune responses. Activation of GPR37 through neuroprotectin D1 (NPD1) and artesunate (ARU) has been shown to decrease serum interleukin-6 (IL-6) levels in WT mice infected with LPS, *Listeria*, and malaria parasites, thereby mitigating inflammation and reducing mortality (Bang et al., 2021). However, it failed to resolve inflammation in GPR37−/− mice (Bang et al., 2021). Research indicates that inflammatory pain, encompassing thermal hyperalgesia and mechanical allodynia, is notably delayed in GPR37−/− mice. These mice demonstrate significantly elevated levels of the pro-inflammatory cytokine IL-1β alongside reduced levels of the anti-inflammatory cytokines IL-10 and transforming growth factor-β (TGF-β) in the skin of their hind paws (Bang et al., 2018). Additionally, GPR37 activation could reduce the degree of cardiac ischemia-reperfusion injury by upregulating the activity of the JNK/PPAR-γ pathway, promoting phagocytic function of cardiac macrophages, M2-type polarization, and expression of anti-inflammatory factors (Zeng et al., 2019).

Acute inflammation and edema frequently occur following injury or infection, initially involving polymorphonuclear neutrophils (PMNs) infiltration. During this process, GPR37 can bind to specialized pro-resolving mediators (SPMs) to exert anti-inflammatory effects (Park et al., 2020), which may be related to macrophage activation. In macrophages, OCN treatment significantly reduces IL-6 and tumor necrosis factor-alpha (TNF-α) induced by LPS while upregulating the expression of anti-inflammatory factors such as IL-10, TGF-β, and Arginase 1 (Arg1). However, in GPR37−/− macrophages, OCN fails to exert the anti-inflammatory effects (Qian et al., 2022). Furthermore, GPR37 has the potential to activate the calcium signaling pathway, leading to an increase in intracellular calcium levels and an enhancement of the phagocytic activity of WT macrophages (Bang et al., 2018; Bang et al., 2021). This process is mainly dependent on Gi protein-coupled signaling. Notably, pretreatment of macrophages with pertussis toxin (PTX), a Gi/o protein inhibitor, abolishes the rapid alterations in intracellular Ca^2+^, cAMP, and pERK levels that OCN triggers in WT macrophages (Qian et al., 2022).

Further studies have linked OCN/GPR37 to neuroinflammation caused by brain dysfunction. In PD models, OCN treatment has been shown to mitigate dopaminergic neuron loss, significantly decreasing the numbers of astrocytes and microglia in the substantia nigra and striatum, along with reductions in TNF-α and IL-1β (Guo et al., 2018). Lower serum GPR37 levels and higher levels of inflammatory markers such as S100β, neuron-specific enolase (NSE), IL-1β, and TNF-α are observed in stroke patients compared to healthy controls. In addition, GPR37 levels are significantly negatively correlated with the NIH Stroke Scale (NIHSS) scores (Li et al., 2024). Animal studies further substantiate the link between GPR37 and neuroinflammation. In a model of fetal alcohol spectrum disorders (FASD) induced by alcohol exposure, significant increases in the expression of pro-inflammatory cytokines, including IL-1β, TNF-α, and chemokine CCL2, were observed in the cerebellum, accompanied by a notable decrease in *GPR37* (Kane et al., 2021). These findings suggest that GPR37 may regulate brain dysfunction by modulating central inflammation.

GPR37−/− mice exhibit significant changes in glial and progenitor cell dynamics in the middle cerebral artery occlusion (MCAO) lesion area. These alterations include a reduction in astrocyte response (McCrary et al., 2019) and increased NPCs and OPCs (Owino et al., 2021). Notably, at earlier time points within 24 h post-stroke, microglial M1 polarization is significantly enhanced, accompanied by elevated levels of pro-inflammatory cytokines like TNF-α, IL-1β, IL-6, and chemokines C-C motif chemokine ligand 2/3 (CCL2/3) (McCrary et al., 2019). CCL2/3 may contribute to the recruitment and infiltration of macrophages into the lesion of brain injury (Ciechanowska et al., 2020; Popiolek-Barczyk et al., 2020). Though these infiltrating macrophages exhibit functional similarities to microglia, they originate from distinct sources (Davies and Miron, 2018). In certain inflammatory conditions, such as multiple sclerosis (MS), macrophages collaborate with microglia, contributing to the pathological processes (Dong and Yong, 2019). Extensive studies in macrophages have established the role of the OCN-GPR37 axis in counteracting peripheral inflammation (Qian et al., 2022). Moreover, findings from GPR37−/− models suggest that GPR37 exerts significant anti-inflammatory effects on the CNS (McCrary et al., 2019). Nevertheless, direct evidence demonstrating the anti-inflammatory function of OCN through GPR37 in the brain remains limited despite the strong plausibility of this mechanism.

While GPR37 is primarily recognized for its substantial anti-inflammatory effects, some individual studies present opposing views. For instance, in glioma, elevated GPR37 is positively correlated with increased infiltration of M2 macrophages, which is associated with a poor prognosis (Liang et al., 2023). In an LPS-induced inflammation model, the enhanced reactivity of enteric glial cells is accompanied by increased GPR37 expression, whereas this response is diminished in GPR37−/− mice (Robertson et al., 2024a) (Table 1)

**TABLE 1:** Effects of GPR37 on inflammation or immune response.

Species	Model 1 (GPR37)	Model 2	Tissue/cell	Phenotype		Treatment (GPR37)	Phenotype after treatment	Reference
				GPR37	Inflammation			
Mice	WT	Peripheral inflammation	Serum	-	↑	↑	Macrophage ablation↓ Inflammation↓ Survival rate↑	Bang et al. (2021)
			Macrophage					
Mice	KO	Inflammatory pain	Hind paw skin	-	↑	-	Delayed pain↑	Bang et al. (2018)
Human	WT	Stroke	Serum	↓	↑	-	NIHSS↑	Li et al. (2024)
Mice	WT	FASD	Cerebellum	↓	↑	-	Inflammation↑	Kane et al. (2021)
Mice	KO	MCAO	Brain	-	↑	-	Inflammation↑	McCrary et al. (2019), Owino et al. (2021)
	WT			↓			-	
Mice	WT	LPS	Serum	↓	↑	↑	Survival rate↑ Inflammation↓	Park et al. (2020), Qian et al. (2022)
			Macrophage					
	KO		Serum	-			Survival rate↓ Inflammation↑	
			Macrophage					
-	-	-	Cardiac macrophage	-	-	↑	M2-type polarization↑	(Zeng et al., 2019)
Mice	KO	LPS	Enteric glial cells	-	↓	-	Reactivity of enteric glial cells↓	Robertson et al. (2024a)

### 3.3 Stress responses

Emerging evidence indicates that GPR37 activation is crucial in protecting primary astrocytes from H_2_O_2_-induced cell death. Notably, this protective function is substantially compromised when endogenous GPR37 expression is downregulated (Meyer et al., 2013). In ischemic stroke models of MCAO, the absence of GPR37 results in elevated apoptosis and autophagy, accompanied by a pronounced increase in infarct size within the damaged region (McCrary et al., 2019). Furthermore, in these regions, GPR37 has been shown to mitigate neuronal apoptosis, promote cell survival, and shrink infarct size through the PI3K/Akt/ASK1 signaling pathway (Yu et al., 2024).

The involvement of GPR37 in cell survival appears to be intricately linked to oxidative stress and endoplasmic reticulum (ER) stress (ERS). A CHIP-Seq experiment in human neuroblastoma cells identified GPR37 as a downstream target gene of NRF1. As a transcription factor, NRF1 is intricately associated with mitochondrial function and oxidative stress, suggesting that GPR37 plays a significant role in the cellular responses to oxidative stress (Satoh et al., 2013). Clinically, elevated levels of GPR37 have been detected in the cerebrospinal fluid (CSF) of patients with medulloblastoma. Moreover, metabolomic profiling reveals that under hypoxic conditions, cyclooxygenase metabolites are almost absent in the CSF, while epoxygenase products and the lipid hormone 12,13-DIHOME, which promotes β-oxidation, are significantly upregulated (Reichl et al., 2020). This increase may reflect a tumor self-regulatory mechanism aimed at reducing inflammation by increasing GPR37 expression, facilitating adaptation to hypoxia, and enhancing invasiveness. While GPR37 overexpression might contribute to tumor progression, it also underscores its protective role in stress-related cellular processes.

A multitude of proteins undergo folding and modification within the ER. When incorrectly folded or improperly assembled, proteins accumulate in the ER lumen, triggering ERS. To mitigate ERS, cells initiate the unfolded protein response (UPR) and activate ER-associated degradation (ERAD), facilitating the retrotranslocation of misfolded proteins to the cytosol for degradation. Consequently, the accumulation of proteins in the cytosol directly results from ER protein aggregation and ERS (Hwang and Qi, 2018). The overexpression of GPR37 further exacerbates protein accumulation in the cytosol, intensifying ERS and promoting neuronal apoptosis (Imai et al., 2001; Marazziti et al., 2009). In PD models, this overexpression activates ERS, enhances autophagy, and selectively degenerates GPR37-expressing neurons by converting LC3-I to LC3-II (Marazziti et al., 2009). Conversely, reducing GPR37 expression can inhibit ERS (Kubota et al., 2006). Dexmedetomidine, an alpha-2 adrenergic receptor (A2AR) agonist, significantly reduces ERS by preventing the accumulation of GPR37 and decreasing the activity of the procaspase-3/CHOP apoptotic pathway in the hippocampus of neonatal mice exposed to buprenorphine (Lin et al., 2021). Two fundamental mechanisms are involved in the role of GPR37 in alleviating ERS. First, the degradation of cytosolic GPR37 represents a pivotal mechanism in mitigating ERS. Research has elucidated that the ubiquitin ligase HRD1 facilitates the ubiquitination and proteasomal degradation of GPR37, thereby attenuating GPR37-mediated ERS and preventing apoptosis (Kaneko, 2016). Second, by promoting the translocation of GPR37 from the cytosol to the plasma membrane (Hertz et al., 2019), the ERS inhibitor 4-phenylbutyric acid effectively reduces the accumulation of misfolded proteins, including GPR37. As a result, it alleviates ERS and mitigates cytosolic protein aggregation and related stress responses (Kubota et al., 2006). In contrast to the potential adverse effects of GPR37 accumulation in the cytosol, the transmission of GPR37 signaling may positively influence ER function. GPR37 facilitates the maturation of LRP6, a glycoprotein essential for maintaining ER homeostasis, thereby ensuring effective Wnt/β-catenin signaling. Additionally, GPR37 protects LRP6 from ER-associated degradation. Consequently, GPR37 mitigates cellular damage induced by ERS (Berger et al., 2017).

**FIGURE 3:**
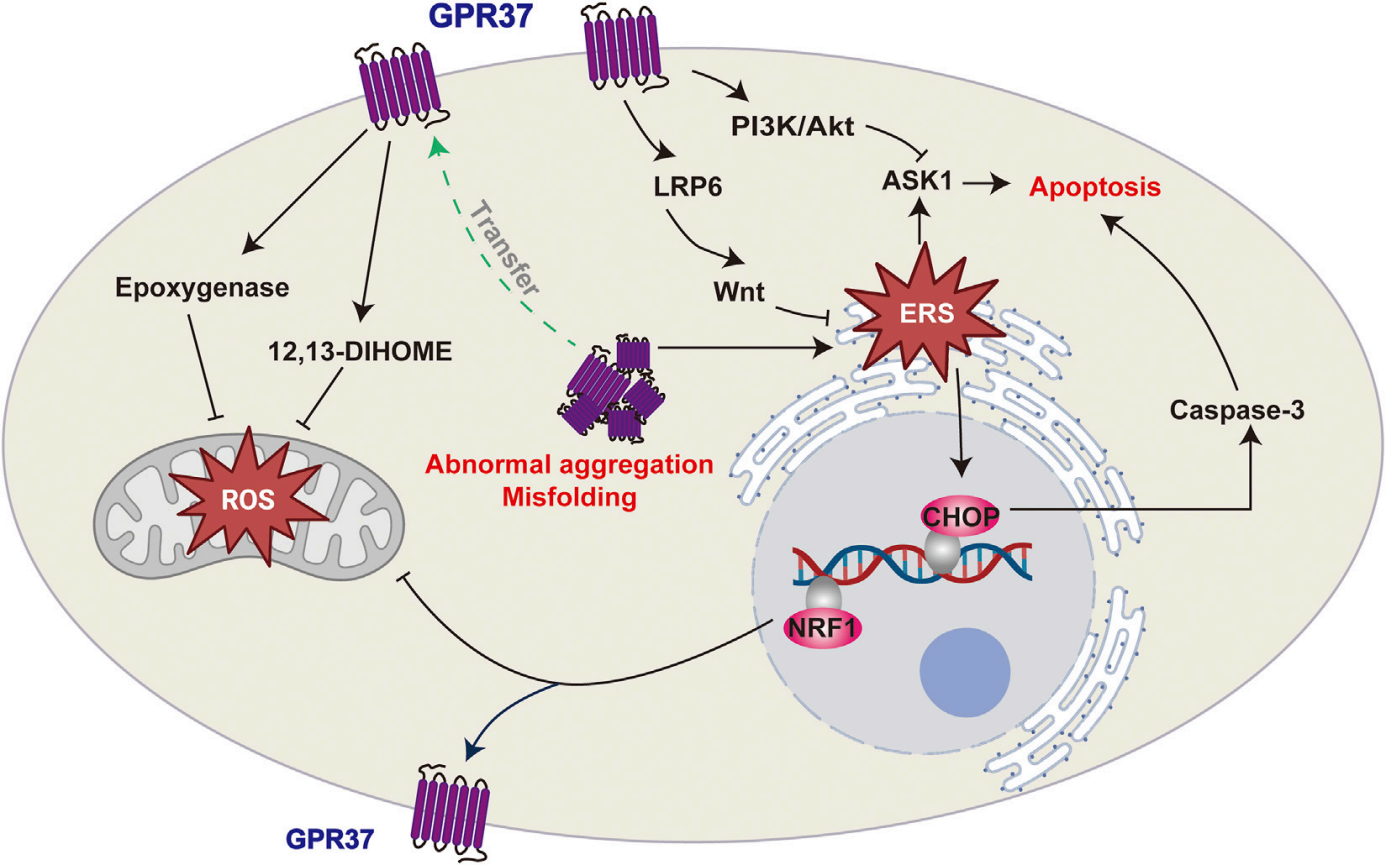
GPR37 and its roles in cellular stress responses. GPR37 regulates oxidative stress and endoplasmic reticulum stress (ERS), which can lead to apoptosis. It influences ROS production in mitochondria and modulates ERS, with misfolding or abnormal aggregation potentially triggering apoptotic pathways.

In summary, GPR37 can potentially alleviate cellular damage induced by oxidative stress or ERS in challenging environments. However, excessive GPR37 expression may exacerbate stress responses and hasten disease progression in specific scenarios, underscoring its dual functionality. This paradox indicates that the functional regulation of GPR37 is highly dependent on the cellular environment and the nature of the stressors. Further investigation is essential to elucidate its therapeutic potential across various pathological conditions (Figure 3).

### 3.4 Neuronal functions

GPR37 was initially identified as related to PD in NDs and was termed the parkin-associated endothelin receptor-like receptor (Pael-R) (Marazziti et al., 2004). Subsequent research has revealed that the function of GPR37 extends beyond PD, playing roles in various physiological processes, including neuroprotection, neurodevelopment, and, notably, synaptic plasticity. In GPR37−/− mice, lower levels of dopamine and dopamine transporter (DAT) have been observed, along with significantly reduced phosphorylation of the AMPA receptor subunit GluA1 and the NMDA receptor subunit GluN2B (Zhang et al., 2020a). These mice also exhibit impaired LTP in striatal neurons, reduced synaptic plasticity, and pronounced motor function deficits (Zhang et al., 2020a). Moreover, the activation of GPR37 by various factors, including OCN, has been shown to exert neuroprotective effects (Meyer et al., 2013; Qian et al., 2021).

The cytoplasmic accumulation of proteins can trigger cytotoxic effects through autophagic overload, stress, and inflammatory responses, collectively leading to cellular dysfunction and potentially accelerating disease progression. Adequately folded and membrane-localized GPR37 exerts neuroprotective effects, whereas misfolded and aggregated GPR37 has been associated with neurodegenerative changes in PD (Zhang et al., 2020a). In a neurotoxicity rat model induced by subcutaneous kainic acid injection, GPR37 was initially strongly expressed in the cytoplasm of Purkinje cells. Still, its levels significantly decreased a few days post-injection (Li et al., 2017). This reduction may be attributed to either increased degradation of cytoplasmic GPR37 or enhanced translocation to the plasma membrane. Inhibition of GPR37 aggregation within the ER or facilitation of its translocation to the plasma membrane may enhance cell viability (Dunham et al., 2009; Lundius et al., 2014). Furthermore, treatment with GM1, a brain-expressed ganglioside, significantly improved the survival of cells stably expressing GPR37 compared to WT cells lacking GPR37 in an MPP + -induced N2a PD cell model (Hertz et al., 2021). These findings indicate that GPR37 is crucial for cell survival. However, PCR analysis showed no significant alterations in GPR37 RNA expression following GM1 treatment, suggesting that the levels of GPR37 expression may not be the determining factor. Instead, forming plasma membrane complexes involving GPR37 may be instrumental in this process (Hertz et al., 2021).

Like other GPCRs, GPR37, located on the plasma membrane, is crucial for signaling recognition and response to external signals, regulating cellular functions, and as a drug target. When it binds to its ligand, such as OCN, GPR37 exerts neuroprotective effects through GPCR-mediated signaling pathways. GPR37 regulates the activity of proteins, including PI3K, Akt, and CaMKII, and promotes Ca^2^⁺ influx via transient receptor potential (TRP) family Ca^2^⁺ channels, facilitating cell mitosis (Rezgaoui et al., 2006). Additionally, GPR37 engages the ERK signaling pathway to promote neuroprotective functions such as OLs differentiation and myelination (Yang et al., 2016). The interaction between GPR37 and membrane proteins is crucial during signal transduction, particularly in modulating synaptic plasticity. While no significant changes in long-term depression (LTD) are observed in striatal and hippocampal neurons in the absence of GPR37, chronic blockade of the A2AR under GPR37−/− conditions enhances LTD and motor sensitization in the striatum (Hertz et al., 2019; Morato et al., 2019). GPR37 also regulates the DAT and dopamine D2 receptors (D2R), influencing dopamine neurotransmission (Leinartaite and Svenningsson, 2017). Loss of GPR37 results in increased DAT expression on the plasma membrane and enhanced DAT-mediated dopamine uptake, which may exacerbate symptoms in patients with PD (Marazziti et al., 2007).

Some studies suggest that downregulation of GPR37 may reduce apoptosis and improve cell survival in PD models, with apoptosis rates decreasing from 39.1% to 29% and cell survival increasing from 56% to 63% when GPR37 is downregulated (Zou et al., 2012). Additionally, though GPR37 inhibits DAT in PD and is beneficial for restoring dopamine signaling, the loss of GPR37 might have positive implications from an addiction treatment perspective. In GPR37−/− mice, the conditioned place preference response to amphetamine and cocaine is significantly reduced. These findings suggest that the absence of the GPR37 affects the reward response to stimulants, which may be beneficial for addiction treatment (Marazziti et al., 2011).

**FIGURE 4:**
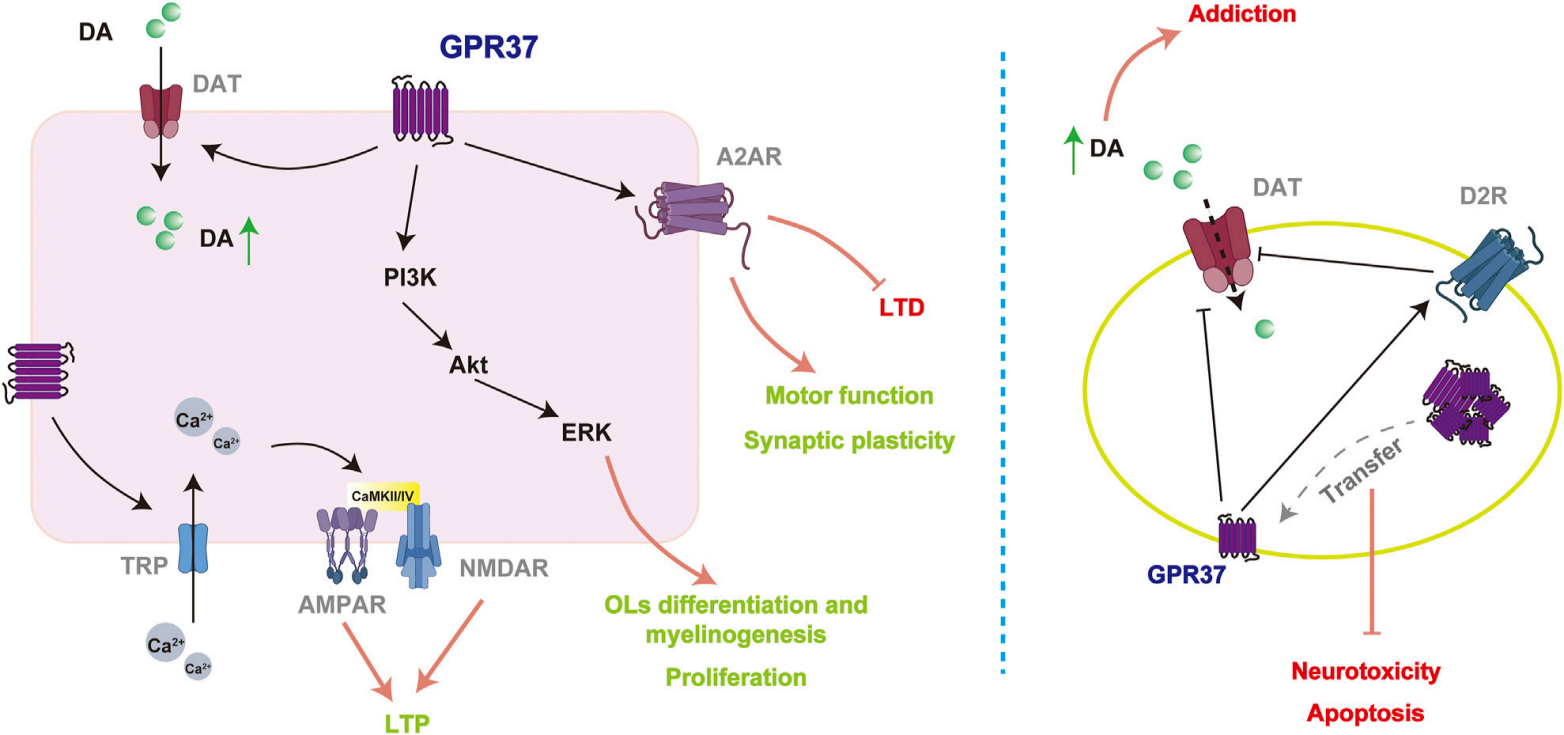
Potential mechanisms of GPR37 in mediating neuroprotective effects. GPR37 enhances synaptic plasticity by regulating multiple ion channels and receptors (left). However, in disease models (right), its mechanisms of action may be reversed entirely compared to physiological conditions, potentially leading to an increase in addictive behaviors.

The role of GPR37 in the nervous system is complex. The expression and localization of GPR37 significantly influence the regulation of the dopamine system, the maintenance of synaptic plasticity, and the response to neuroprotective factors such as OCN. When GPR37 is translocated to the plasma membrane and interacts with its ligands, it can exert neuroprotective effects through GPCR signaling pathways, including regulating the PI3K/Akt and ERK signaling pathways. However, dysfunction of GPR37 or its abnormal accumulation within cells can weaken its neuroprotective functions and is associated with developing various NDs (Figure 4).

## 4 OCN/GPR37 and NDs

Lower OCN levels are associated with alterations in brain microstructure (Puig et al., 2016). Mutations in the runt-related transcription factor 2 (RUNX2), which acts as an upstream regulator of OCN, result in cleidocranial dysplasia, frequently presenting as cognitive impairment (Takenouchi et al., 2014). In NDs, research on OCN has primarily focused on PD and various forms of dementia. In PD rat models, CSF OCN levels were significantly reduced, while OCN treatment mitigated the loss of tyrosine hydroxylase, a key enzyme involved in DA synthesis within the nigrostriatal pathway (Guo et al., 2018). Additionally, OCN was shown to reduce apoptosis of dopaminergic neurons in PD mouse models, alleviate neurotoxicity, and improve motor function impairments by modulating the Akt/glycogen synthase kinase 3beta (GSK3β) signaling pathway (Hou et al., 2021). A Mendelian randomization study explored the causal relationship between OCN and various forms of dementia, including AD, PD, Lewy body dementia (LBD), and vascular dementia (VD). The findings indicated that OCN exerts a significant impact on dementia, with its potential protective effect being more pronounced in AD compared to other types (Liu et al., 2023). Furthermore, animal studies demonstrated that intraperitoneal injection of OCN reduced Aβ levels in the hippocampus and cortex of AD mouse models, enhanced the power of high gamma band in medial prefrontal cortex, and improved anxiety-like behavior and cognitive dysfunction (Shan et al., 2023).

Remarkably, OCN supplementation has been demonstrated to ameliorate diabetes-associated cognitive deficits in a dose-dependent manner, an effect abrogated by the administration of Akt inhibitors (Zhao et al., 2024). In AD, OCN enhances cognitive function by reducing Aβ accumulation and upregulating glycolysis in glial cells (Shan et al., 2023). Moreover, alterations in glucose metabolism across multiple brain regions indicate the abnormal distribution of α-synuclein aggregates, contributing to the progression of PD (Scholefield et al., 2023). In Huntington’s disease (HD) models, neuropathological alterations and motor deficits are accompanied by the progression of glucose intolerance and tissue wasting (Duan et al., 2003; Patassini et al., 2016). These findings indicate that OCN may play a crucial role in modulating cognitive function associated with aging and NDs, potentially through its influence on glucose metabolism.

The GPR37 is integral to the pathological processes underlying various brain disorders, with its deletion shown to impair oligodendrocyte function and elevate susceptibility to demyelinating diseases, notably MS (Smith et al., 2017). Additionally, proteomic analyses of brain tissue have identified that the s100 calcium-binding protein A5 (S100A5), implicated in mood disorders, exhibits marked alterations in the absence of GPR37, underscoring GPR37’s potential role as a biomarker for neurological damage (Nguyen et al., 2020). Interestingly, a GPR37-Del321F mutation was detected in the unaffected father of an individual with autism spectrum disorder (ASD), while the GPR37-R558Q mutation was present in the affected brother and the unaffected mother (Fujita-Jimbo et al., 2012). The pathophysiological impact of the R558Q mutation is likely due to its interference with GPR37’s synaptic localization, as it prevents co-localization with synaptic scaffolding proteins multi-PDZ domain protein 1 (MUPP1) and contactin-associated protein-like 2 (CASPR2), leading to GPR37 retention within the endoplasmic reticulum and a consequent increased ASD risk (Tanabe et al., 2015).

Although the extent to which GPR37 mediates the functions of OCN remains uncertain, several studies have shed light on the complex role of GPR37. Similar to OCN, GPR37 is involved in the regulation of DA levels. In GPR37−/− mice, striatal DA levels were reduced to 60% of those in control groups. Conversely, in GPR37-overexpressing mice, striatal levels of 3,4-dihydroxyphenylacetic acid and vesicular DA were elevated (Imai et al., 2007). Additionally, GPR37−/− mice displayed dopaminergic neuron loss, LTP deficits, and increased susceptibility to neurotoxicity induced by 6-hydroxydopamine (Zhang et al., 2020a) along with pronounced anxiety- and depression-like behaviors (Mandillo et al., 2013). Notably, under pathological conditions, particularly in NDs, GPR37 activation may aggravate disease progression. Overexpression of GPR37 has been found to increase the vulnerability of dopaminergic neurons to chronic DA toxicity and promote apoptosis (Imai et al., 2007; Kitao et al., 2007). In contrast, the downregulation of GPR37 enhanced cell survival in PD models (Zou et al., 2012).

GPR37 shows potential as a biomarker for NDs. Both the correlations and distinctions in the unique processing mechanisms of GPR37 across various types of NDs (Argerich et al., 2024). In the striatum of AD patients, GPR37 levels were significantly elevated, though no corresponding increase was observed in CSF. In contrast, PD patients exhibited significantly higher levels of GPR37 in the CSF, suggesting that GPR37 might serve as a biomarker for PD progression rather than AD. Notably, this elevation was restricted to patients with slow progressive PD (Morato et al., 2021; Argerich et al., 2024). Beyond NDs, GPR37 expression also varies across psychiatric conditions. It was markedly downregulated in major depressive disorder but significantly upregulated in bipolar disorder (Tomita et al., 2013). Additionally, GPR37 plays a pivotal role in myelination, making it relevant to MS, a disorder characterized by progressive axonal demyelination in the central nervous system. These findings offer valuable insights into the roles of OCN and GPR37 in disease pathogenesis and progression, underscoring the importance of further investigation into their mechanisms.

## 5 Conclusion and prospective

Current evidence underscores the predominantly beneficial role of OCN in regulating brain function. This effect is linked to several signaling pathways, including RhoA/GTPase, PI3K/Akt/ASK1, ERK, Wnt/β-catenin, IP3/CaMKII, and cAMP/PKA. Under most physiological conditions, GPR37 serves a complementary or mediating role in enhancing the effects driven by OCN. The absence of either OCN or GPR37 results in excessive myelination, with GPR37 mediating the effects of OCN (Qian et al., 2021). In inflammatory responses, both OCN and GPR37 have predominantly demonstrated anti-inflammatory effects (McCrary et al., 2019; Qian et al., 2021), though the anti-inflammatory role of OCN in the central nervous system has yet to be fully validated. Additionally, both OCN and GPR37 display neuroprotective properties in NDs. Nevertheless, GPR37 may also display roles that diverge from OCN. For instance, the intracellular accumulation of GPR37 has been linked to aggravated stress responses (Marazziti et al., 2009). Furthermore, GPR37 is highly expressed in peripheral inflammatory models (Robertson et al., 2024b) and certain NDs, where it has been identified as a potential prognostic biomarker (Morato et al., 2021; Argerich et al., 2024).

While evidence has supported a connection between OCN and GPR37, their multi-receptor and multi-ligand interactions warrant further investigation to clarify whether their effects are synergistic or divergent. In addition to OCN, GPR37 binds a range of ligands including head activator (Rezgaoui et al., 2006), prosaposin (Bhattacharya et al., 2023; Yu et al., 2024), regenerating islet-derived family member 4 (Wang et al., 2016), NPD1(Bang et al., 2018), and the agonist ARU (Bang et al., 2021). The diversity of ligands increases the complexity of GPR37 in brain cognitive function and may explain the dual roles of GPR37 under different physiological and pathological conditions. Understanding the effects of these ligands will provide a theoretical foundation for developing novel therapeutic strategies based on the OCN/GPR37 axis, potentially achieving significant breakthroughs in treating cognitive dysfunctions and NDs.

Future research should prioritize exploring the specific signaling pathways and molecular mechanisms through which OCN affects GPR37, particularly its dual roles in different brain regions and pathological states. Understanding how to regulate OCN levels and GPR37 activity is crucial for future studies. Exercise is currently recognized as the most effective non-invasive strategy for enhancing circulating and brain OCN levels, with evidence suggesting that this elevation is independent of exercise modality, duration, gender, or age (Chahla et al., 2015; Armamento-Villareal et al., 2020; Hiam et al., 2021; Mohammad Rahimi et al., 2021; Koltun et al., 2024). However, further investigation is required to identify the specific exercise type that optimally promotes OCN secretion and GPR37 activation. Moreover, the recent discovery of GPR158 as an additional central receptor for OCN raises the possibility of functional overlap with GPR37 (Khrimian et al., 2017). Elucidating the relationship and functional differentiation between these two receptors is a critical area of ongoing research.

## Glossary

A2AR: alpha-2 adrenergic receptor
AD: Alzheimer’s disease
Arg1: Arginase 1
ARU: artesunate
ASD: autism spectrum disorder
Aβ: amyloid-beta
BBB: blood-brain barrier
CCL2/3: chemokines C-C motif chemokine ligand 2/3
CNS: central nervous system
CSF: cerebrospinal fluid
Cspg4: chondroitin sulfate proteoglycan 4
D2R: D2 receptors
DAT: dopamine transporter
Dcx: doublecortin
ERS: endoplasmic reticulum stress
FASD: fetal alcohol spectrum disorders
GlialCAM: glial cell adhesion molecule
GnRH: gonadotropin-releasing hormone
GPR37: G protein-coupled receptor 37
LPS: lipopolysaccharide
LRP6: lipoprotein receptor-related protein 6
LTD: long-term depression
MAP2: Microtubule-associated protein 2
MBP: myelin basic protein
MCAO: middle cerebral artery occlusion
MLC: megalencephalic leukoencephalopathy with subcortical cysts
NDs: neurodegenerative diseases
NPCs: neural progenitor cells
NPD1: neuroprotectin D1
NSE: neuron-specific enolase
OCN: Osteocalcin
OECs: olfactory ensheathing cells
OLs: oligodendrocytes
OPCs: oligodendrocyte precursor cells
Pael-R: parkin-associated endothelin receptor-like receptor
PD: Parkinson’s disease
PLP1: proteolipid protein 1
PMNs: polymorphonuclear neutrophils
PTX: pertussis toxin
SPMs: specialized pro-resolving mediators
TGF-β: transforming growth factor-β
TNF-α: tumor necrosis factor-alpha
TRP: transient receptor potential
VIM: vimentin
